# Concentration of Antioxidant Compounds from *Calendula officinalis* through Sustainable Supercritical Technologies, and Computational Study of Their Permeability in Skin for Cosmetic Use

**DOI:** 10.3390/antiox11010096

**Published:** 2021-12-30

**Authors:** Raquel Mur, Elisa Langa, M. Rosa Pino-Otín, José S. Urieta, Ana M. Mainar

**Affiliations:** 1 GATHERS Group, Aragón Institute of Engineering Research (I3A), Universidad de Zaragoza, c/. Mariano Esquillor s/n, 50018 Zaragoza, Spain; 649396@unizar.es (R.M.); urieta@unizar.es (J.S.U.); 2 Campus Universitario Villanueva de Gállego, Universidad San Jorge, Autovía A-23 Zaragoza-Huesca Km. 299, 50830 Villanueva de Gallego, Spain; elanga@usj.es (E.L.); rpino@usj.es (M.R.P.-O.)

**Keywords:** supercritical antisolvent fractionation, ferulic acid, caffeic acid, chlorogenic acid, permeability skin

## Abstract

The growing interest in the cosmetic industry in using compounds of natural and sustainable origin that are safe for humans is encouraging the development of processes that can satisfy these needs. Chlorogenic acid (CHA), caffeic acid (CAF) and ferulic acid (FA) are three compounds widely used within the cosmetic industry due to their functionalities as antioxidants, collagen modifiers or even as radiation protectors. In this work, two advanced separation techniques with supercritical CO_2_ are used to obtain these three compounds from *Calendula officinalis*, and these are then evaluated using a computational skin permeability model. This model is encompassed by the COSMO-RS model, the calculations of which make it possible to study the behaviour of the compounds in the epidermis. The results show that both CAF and FA are retained in the stratum corneum, while CHA manages to penetrate to the stratum spinosum. These compounds were concentrated by antisolvent fractionation with super-critical CO_2_ using a Response Surface Methodology to study the effect of pressure and CO_2_ flow rate. CHA, CAF and FA were completely retained in the precipitation vessel, with concentrations between 40% and 70% greater than in the original extract. The conditions predicted that the optimal overall yield and enrichment achieved would be 153 bar and 42 g/min.

## 1. Introduction

In recent years, there has been great interest in the cosmetic industry, among others, in including compounds of natural and sustainable origin in the formulation of products. This is due to, on the one hand, growing general concern related to the possible harmful effects of synthetic compounds and, on the other hand, awareness regarding the responsible consumption of these sustainable products, causing an increase in their demand. Additionally, the traditional use of plants as natural remedies, or even as cosmetics, makes them ideal candidates from which to obtain compounds that meet these requirements.

*Calendula officinalis L*., also known as marigold, is an aromatic herbaceous plant that belongs to the genus Calendula (*Asteraceae*), native to the Mediterranean countries. This genus includes approximately 25 species, of which *C. officinalis* is the only one used extensively clinically worldwide [1], traditionally as a skin remedy for dermatological problems, such as inflamed skin, redness, minor burns, or ulcers, as well as for acne or fungal eruptions [2]. Over the years, studies have demonstrated these properties, which have been duly collected in various monographs such as those by the European Scientific Cooperative On Phytotherapy (ESCOP), the European Medicines Agency (EMA) or the World Health Organization (WHO), where wound healing and anti-inflammatory actions stand out [3,4,5]. In fact, different organizations that control and regulate substances for human consumption, such as the Food and Drug Administration (FDA) or the European Chemicals Agency (ECHA), have several guidelines to regulate extracts obtained from *C. officinalis* (Directive 2004/24/EC, CFR Title 21 from FDA, etc.). They also have Chemical Abstracts Service (CAS) registry numbers (84776-23-8/70892-20-5) and European INventory of Existing Commercial chemical substances (EINEC) numbers (283-949-5/615-192-7). Regarding cosmetic use, *C. officinalis* is especially valuable for its assistance in cell rejuvenation, wound healing, inflammation reduction, soothing, and skin softening [6]. According to the EU cosmetic ingredient database, CosIng, the extracts of this plant have several functions as a cosmetic: skin conditioning, emollient, skin protection, fragrance, perfuming, and even flavouring [7,8,9]. Furthermore, it has been found that calendula oil cream can be used to protect the skin from UV radiation in the form of sunscreen cream and to maintain the natural pigmentation of the skin [10].

The potential bioactivity of *C. officinalis* has so far focused on its composition, which has been studied extensively. Important components of these plants include triterpenic saponins, flavonoids, carotenoids, sesquiterpenoids and polyphenols, among others, responsible for, for example, antimicrobial [2,11], wound and burn healing [12,13], photoprotective [14], anti-inflammatory and analgesic, [15,16] cytotoxic and immunological [17,18], and even neuroprotective [19] and cardiovascular activities [20]. Within the group of polyphenols, ferulic acid (FA), caffeic acid (CAF) and chlorogenic acid (CHA) stand out due to their high antioxidant capacity [21,22,23], their anti-wrinkle and anti-aging effects [24,25,26], and the ability to modify collagen properties [27]. Furthermore, a potential role in radiation protection and photooxidation has also been reported [28,29,30]. All this makes them compounds of great interest for the cosmetic industry, which already has formulations that include them [31], and for this reason they will be the subject of greater interest in this work.

On the other hand, plant extracts are usually obtained using traditional techniques such as hydrodistillation or Soxhlet working at high temperatures, which can cause the degradation of the bioactive compounds of interest. In addition, these are not sustainable processes due to possible environmental contamination and the use of organic solvents that can be harmful to human health. In this regard, the use of advanced separation technologies such as that using supercritical fluids, especially supercritical carbon dioxide (sc-CO_2_), is of great interest for the extraction of bioactive compounds from natural sources [32,33]. This is due to its properties, since CO_2_ is harmless and non-flammable, and its supercritical conditions (Pc = 74 bar, Tc = 31 °C) are moderate enough to avoid the degradation of compounds [32]. Furthermore, it can be easily removed by reducing the pressure, becoming a gas, which generates a final extract with no residual solvent presence [34].

There are various techniques that work with sc-CO_2_. Supercritical fluid extraction (SCE) is one of the most widely used and studied techniques for the extraction of certain compounds like monoterpenes, sesquiterpenes, phenols, etc. [35]. The most studied parameters in this technique are the temperature, pressure, CO_2_ flow rate, particle size of the sample, and the use of co-solvents that modify the polarity of CO_2_ and allow the extraction of other compounds different from the above-mentioned ones [35,36]. Regarding the extraction of *C. officinalis*, there have been numerous studies applying this technique to obtain compounds such as triterpenoid esters [37], triterpene triols [38], or its essential oil [39,40]. Another promising technique is supercritical antisolvent fractionation (SAF), which makes it possible to precipitate and concentrate bioactive compounds selectively from a solution, while the rest remain dissolved [41]. For this purpose, the solution is pumped and sprayed into a vessel, where it converges with sc-CO_2_ in such a way that the insoluble compounds in this new mixture (solvent + sc-CO_2_) precipitate, while the still soluble ones are collected in a second vessel as a dissolution. In this way, two fractions enriched in different compounds are obtained. This technique has been used to obtain fractions rich in compounds of interest having various bioactivities such as biopesticides [42], or antiproliferative [43], antimicrobial [44], or antioxidant activity [45].

Taking into account the above, one of the objectives of this work is to obtain, in a sustainable way and by combining two advanced separation techniques, an extract enriched in antioxidant compounds from Calendula officinalis. To do this, first, an extraction with supercritical CO_2_ will be carried out to defat the plant material and facilitate obtaining the antioxidant substances. After maceration in ethanol, a study of the influence of pressure (80–160 bar) and of the CO_2_ flow rate (10–60 g/min) on the SAF process will be performed. This will be performed using a Response Surface Methodology (RSM) based on Central Composite Design (CCD), which will provide the values for the studied magnitudes and the sequence to follow when doing the experiments. The compounds chosen to be concentrated on were FA, CHA and CAF, due to their antioxidant properties [21,22,23].

On the other hand, the fact that a compound is of natural or synthetic origin does not affect the possibility that it may have harmful or beneficial effects on people’s health, so it is necessary to study its interaction with human tissues. In vivo and in vitro tests are expensive and difficult to perform, in addition to the fact that there are several regulations that strongly recommend or require the use of alternatives to animal studies (EU REACH regulation) or that even prohibit the marketing of cosmetics with ingredients tested on animals (EU Cosmetics Regulation EC 1223/2009) [46]. In this regard, in silico experimentation is of great interest, because it makes the study of the interaction of solutes with biological membranes possible, and therefore, their possible toxicity. There are various predictive models for the bioavailability of solutes based on quantitative structure–permeability relationship (QSPR) models, molecular dynamics (MD) simulation, or mechanistic models derived from first principles such as mass balance, relying on additional assumptions such as Fick’s laws of diffusion [47].

In this research framework, in silico research on the interaction of solutes with biomembranes, the study of molecular mechanisms behind skin permeation stands out, since it is a tool that makes it possible to identify inappropriate ingredients that may be dangerous in a cosmetic composition [48,49]. One of the most innovative models is the one proposed by Schwöbel et al. [46] which is based on the use of the Conductor-like Screening model for Real Solvents (COSMO-RS). From the extensions of this model—COSMOperm, COSMOmic and COSMOplex—a computational skin model that makes it possible to calculate the permeability of different compounds in it is generated. This model was applied to study the interaction of the tracked compounds of *C. officinalis*, CHA, CAF, FA, with the skin and thus to calculate their permeabilities.

## 2. Materials and Methods

### 2.1. Plant Material

*Calendula officinalis* L. flowers were collected from an ecological cultivation in Huesca (Spain) by Valentia, an association where people at risk of social exclusion work. The plant material was dried in a dryer at room temperature. Dried *Calendula* flowers had an 11.29 ± 0.48 wt% humidity content, determined ten times with a *Sartorious* model MA 40 *Moisture Analyzer* (Goettingen, Germany). The plant material was ground with an electric grinder and sieved with a vibratory sieve shaker (*CISA* model BA 300N, Barcelona, Spain). The average diameter of particles was 0.5 mm. This was adjusted to a normal distribution according to ASAE S319.3 from the American National Standards Institute [50].

### 2.2. Chemicals and Reagents

The CO_2_ (99.9%) used in SCE and SAF was purchased from Carburos Metálicos (Zaragoza, Spain) and ethanol (99.9%) for maceration was obtained from VWR Chemicals (Barcelona, Spain). The solvents used for HPLC analysis were ethanol (PanReac AppliChem 99.9%, Barcelona, Spain), formic acid (PanReac AppliChem 98%, Barcelona, Spain), acetonitrile (Scharlau 99.9%, Barcelona, Spain) and MilliQ water (18.2 MΩ·cm, Zaragoza, Spain). The HPLC standards ferulic acid (≥99%) and caffeic acid (≥98%) were purchased from Sigma-Aldrich (Madrid, Spain), whereas chlorogenic acid (97.1%) was procured from European Pharmacopoeia Reference Standard (Madrid, Spain).

### 2.3. Supercritical CO_2_ Extraction (SCE)

In a first stage, the plant material was defatted with supercritical CO_2_ in a Waters laboratory scale plant (SFE-1000F-2-FMC10 System, Pennsylvania, USA). Its scheme has been published previously [51] and its main components consist of an extraction vessel of 1 L (E), and two 0.5 L collectors (C1, C2). All three vessels were jacketed to maintain a constant temperature. The CO_2_ stored in a bottle was passed through a cooling bath (CB) to keep it liquid and then propelled through a pump (P2) towards E. A heat exchanger (HE) was used to ensure that the CO_2_ passing into E was above the critical temperature. Flow rate and temperature were automatically controlled, as was the pressure in E. The pressure in C1 and C2 was controlled by manual back pressures (MBPR).

The procedure is the same as that performed in previous works [51]. The same proportion of plant material/inert glass bead was used (100 g/200 g), which allows a better CO_2_–solid contact, and therefore facilitates better extraction. The complete extraction process consisted of 4 static–dynamic cycles and the extraction conditions were 350 bar, 40 °C in E, 90 bar, 45 °C in C1 and 30 bar and 30 °C in C2. The CO_2_ flow rate was 60 g/min. Once the machine was depressurized, the extracts collected from C1 and C2 and the plant material recovered from E were stored in a freezer for further experiments and analysis.

The supercritical extraction yield (*Y*_SCE_) was calculated through Equation (1):

(1) $$Y_{\mathrm{SCE}}\left(\mathrm{wt}\%\right)=\left(\frac{mass\;{\left(g\right)}_{C1}+mass\;{\left(g\right)}_{C2}}{mass\;{\left(g\right)}_{\mathrm{vegetal}\;\mathrm{material}}}\right)\cdot 100$$

where *mass(g)*_C1_ and *mass(g)*_C2_ are the mass in grams of the extract deposited in collectors C1 and C2, respectively, of the SCE device, and *mass(g)*_vegetal material_ is the initial mass in grams of the dried plant material loaded in the extractor.

### 2.4. Maceration and Supercritical Antisolvent Fractionation (SAF) Processes

Maceration with ethanol (EtOH) was performed to obtain the polar and active compounds of *C. officinalis* flowers. A 3 L volume of the solvent was used to macerate 300 g of vegetal material, previously defatted with SCE, for 48 h at room temperature (25 °C). Then, a rotatory evaporator (Büchi R-200, Flawil, Switzerland) was used to remove the solvent and obtain the dry extract. Equation (2) indicates the formula to calculate the maceration yield:

(2) $$Y_{\mathrm{EtOH}}\left(\mathrm{wt}\%\right)=\left(\frac{mass_{\mathrm{extract}}\;\left(g\right)}{mass_{\mathrm{vegetal}\;\mathrm{material}\;}\left(g\right)\;}\right)\cdot 100$$

where *mass*_plant extract_ (*g*) was the mass of the solvent-free extract obtained and *mass*_vegetal material_ (*g*) was the initial mass, in grams, of plant material subjected to maceration and on which the SCE process was applied.

**Figure 1:**
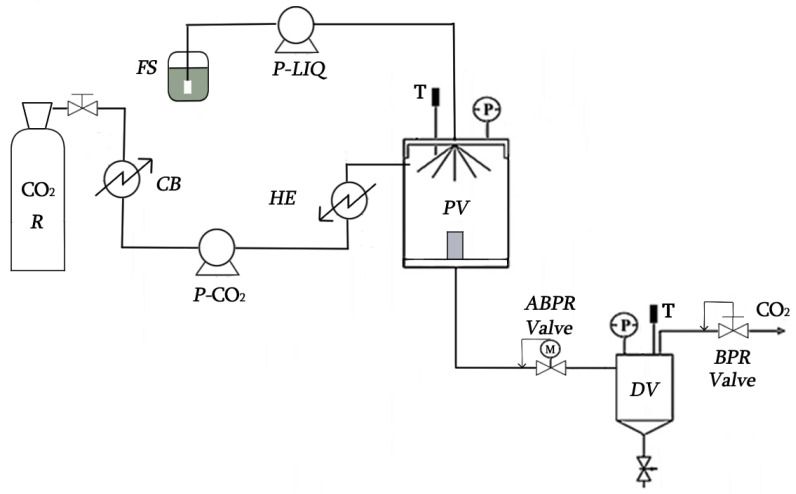
Scheme of the SAF equipment. Feed solution reservoir (FS); FS pump (P-LIQ); CO_2_ reservoir (R); cooling bath (CB); CO_2_ pump (P-SCF); heat exchanger (HE); precipitation vessel (PV); downstream vessel (DV); automatic back pressure regulator (ABPR); back pressure regulator (BPR); thermopar (T); Bourdon (P).

To prepare the feed solution (FS) for each of the SAF experiments, the extract obtained (without solvent) was redissolved in ethanol at 3% (wt%) and filtered through Cellulose Acetate filters with a pore size of 0.22 μm. SAF experiments were carried out by a laboratory-scale plant (Waters, Pennsylvania, USA), the scheme of which is represented in Figure 1. The plant was equipped with a CO_2_ pump (P-SCF), an FS pump (P-LIQ), a 0.5 L precipitation vessel (PV), and a 0.5 L downstream vessel (DV). Temperatures and flow rates of both CO_2_ and FS were automatically controlled, as was pressure in PV (ABPR), but pressure in DV was controlled by manual back pressure regulator (MBPR) [52].

A typical SAF experiment starts when CO_2_ is pumped by P-SCF through a heat exchanger (HE) to PV and the rest of the plant. Once the selected condition of pressure, temperature and CO_2_ flow rate in the system have stabilised, FS is pumped into the PV through an injector (nozzle Ø = 100 mm). In this vessel, the insoluble compounds in the supercritical mixture (CO_2_ + EtOH) precipitate, while the soluble compounds are gathered in DV. Once the flow of FS has finished, 30 mL of pure ethanol is pumped to draw the remaining FS from the pipes. Finally, a flow of CO_2_ is maintained to eliminate the residual ethanol from the solid precipitated in PV. The conditions used were selected on the basis of the previous knowledge of the research group: to avoid thermal degradation, temperature in PV was fixed at 40 °C; to guarantee the supercritical state of the mixture (CO_2_ + EtOH), the FS flow rate and the FS concentration were fixed at 0.45 mL/min and 3% (wt%), respectively, for all experiments [53]. Then the pressure in PV and the CO_2_ flow rate were studied and varied between 80 and 160 bar and 10 and 60 g/min, respectively.

Equation (3) was used to calculate mass recovery yields for the precipitation (PV) and the downstream (DV) vessel fractions, *Y*_PV_ and *Y*_DV_, respectively, of the SAF device:

(3) $$Y_{i}\left(\mathrm{wt}\%\right)=\left(\frac{{mass\;\mathrm{fraction}\;\mathrm{collected}}_{i}}{\mathrm{mass}\;\mathrm{of}\;\mathrm{extract}\;\mathrm{in}\;\mathrm{FS}}\right)\cdot 100$$

where i refers to the vessel from which the mass is collected: PV or DV. The overall recovery yield of the process, *Y*_SAF_, was calculated using Equation (4):

(4) $$Y_{\mathrm{SAF}}\left(\mathrm{wt}\%\right)=Y_{\mathrm{PV}}\left(\mathrm{wt}\%\right)+Y_{\mathrm{DV}}\left(\mathrm{wt}\%\right)$$

### 2.5. HPLC Analysis

**Figure 2:**
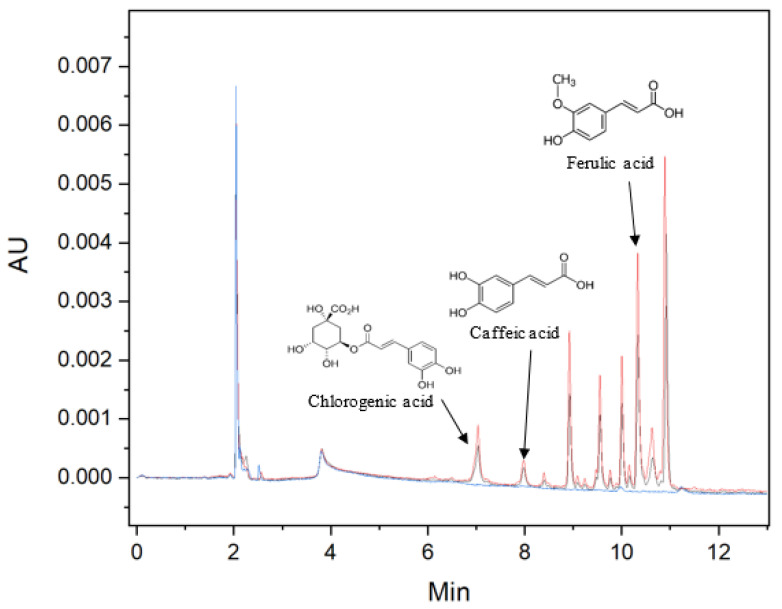
Chromatogram. Peak 1 CHA (T_R_ = 7.04 min, λ = 324 nm), peak 2 CAF (T_R_ = 7.98 min, λ = 324 nm) and peak 3 FA (T_R_ = 10.33 min, λ = 324 nm). PV fraction: red line; FS fraction: blue line.

The equipment used to analyse the SAF fractions of *Calendula officinalis* (FS, PV and DV) was a HPLC *Waters^®^ Alliance 2695* (Milford, MA, USA) with a *PDA Waters^®^ 2998* (Massachusetts, USA) detector working at λ = 324 nm. A reverse column C18 was used (*CORTECS^®^* C18 2.7 μm, 4.6 × 150 mm, (Waters^®^, MA, USA) in conjunction with a pre-column CORTECS^®^ Pre-column VanGuard C18 2.7 μm (2.1 × 5 mm) (Waters^®^, MA, USA). For the chromatographic analysis, a gradient elution at 30 °C was applied with formic acid (0.1%): Milli-water: acetonitrile. The gradient was 10:90:0 to 10:40:50 for 15 min, 10:40:50 to 10:10:80 for 5 min, 10:10:80 to 10:90:0 for 5 min and 10:90:0 for another 5 min. The flow rate applied was 0.8 mL/min. For each sample 100 ppm was prepared in SAF fractions (FS, PV and DV) and filtered through a 0.2 μm filter (*GH Polypropylene membrane ACRODISC* 13 mm, Waters^®^, MA, USA) [51]. CHA, CAF and FA standards were used to build the calibrations curves, and the conditions for analysing them were the same. In Figure 2, retention times can be observed for each compound: CHA 7.04 min, CAF 7.98 min and FA 10.33 min. The analyses of both the samples and the standards were performed in triplicate.

Equation (5) was used to calculate the concentration of a compound in each sample analysed:

(5) $$C_{i/j}\left(\mathrm{wt}\%\right)=\left(\frac{mass\;of\;compound\;i\;in\;fraction\;j}{mass\;of\;fraction\;j}\right)\cdot 100$$

where i is the considered antioxidant (CHA, CAF, FA) and j refers to the fraction in which the compound was analysed, i.e., FS, PV or DV. Once *C*_i/j_ had been obtained, the enrichment ratio $E_{i/j}$ was obtained for each compound using Equation (6):

(6) $$E_{i/j}=\frac{C_{i/j}}{C_{i/\mathrm{FS}}}$$

where, again, i refers to the compound analysed (CHA, CAF, FA) and j is PV or DV. This equation can be modified to calculate how much the PV or DV fractions are enriched in antioxidant compounds. Equation (7) shows how the enrichment ratio of all compounds analysed in PV fraction can be calculated:

(7) $$E_{\mathrm{ALL}/\mathrm{PV}}=\frac{C_{\mathrm{CHA}/\mathrm{PV}}+C_{\mathrm{CAF}/\mathrm{PV}}+C_{\mathrm{FER}/\mathrm{PV}}}{C_{\mathrm{CHA}/\mathrm{FS}}+C_{\mathrm{CAF}/\mathrm{FS}}+C_{\mathrm{FER}/\mathrm{FS}}}$$

### 2.6. Experimental Design and Statistical Analysis

One of the aims in this work is to evaluate the influence of pressure and CO_2_ flow rate on the SAF process and then to optimize the conditions to obtain the best results on yield and enrichment ratios. To statistically study this, a response surface methodology (RMS) based on central composite design (CCD) was used. The values were set between 80 and 160 for pressure in PV, *X*_P_, and 10–60 g/min for CO_2_ flow rate, *XQ*_CO_2__. The rest of the parameters in the experiments—temperature in PV and DV, pressure in DV, and FS flow rate—were constant and were set according to previous experience to ensure the supercritical conditions of the CO_2_–ethanol mixture [51].

The mathematical model of a two variable CCD makes it possible to correlate, through Equation (8), a dependent variable, *Y*, with some independent variables, *X*_i_ and *X*_j_:

(8) $$Y=\beta_{0}+\overset{2}{\underset{i=1}{\sum }}\beta_{i}X_{i}+\overset{2}{\underset{i=1}{\sum }}\beta_{\mathrm{ii}}X_{i}^{2}+\overset{2}{\underset{i\neq j=1}{\sum }}\beta_{\mathrm{ij}}X_{i}X_{j}$$

where *β*_0_ is a constant coefficient, *β*_i_ is the linear coefficient, *β*_ii_ is the quadratic coefficient, and *β*_ij_ is an interaction coefficient. In this work, the dependant variable refers to both the yields of the SAF process (*Y*_PV_, *Y*_DV_ and *Y*_SAF_) and the enrichment ratios of the tracked compounds (CHA, CAF and FA), while the independent variables, which are the variables under study, are pressure (*X*_P_) and CO_2_ flow rate (*XQ*_CO_2__).

The software used to perform the CCD design was *Minitab^®^ 18*. This design provides 13 random experiments, 5 of which are replicates of the central conditions according to the range levels of the variables used, shown in Table 1. This software was also used to determinate the values of each coefficient, *β*, in Equation (8) and their significance (when *p* < 0.05). Finally, *Minitab^®^ 18* was also used to obtain the optimal conditions for the maximum overall recovery yield (*Y*_SAF_) and maximum enrichment ratio of the bioactive compounds (CHA, CAF, FA) (*Y*_ALL/PV_).

### 2.7. Application of the Skin Model

COSMO-RS is a continuum solvation model that makes it possible to calculate thermodynamic properties, generating a three-dimensional distribution of surface polarization charge-densities, σ, from optimized 3D structures of molecules [53,54]. This σ is used, by means of an efficient statistical thermodynamic model of pairwise molecular surface interactions, to calculate the σ-potential, which gives the chemical potential of a surface segment of polarity in a particular solvent. Then, those segments are added and corrected by a combinatorial term to calculate the chemical potential of a compound in a pure or mixed solvent [53]. This can be applied, for example, to calculate the partition coefficient, K, of a solute between two liquid phases [55].

COSMOperm is an extension of the COSMO-RS model that applies these calculations in inhomogeneous systems such as biomembranes. The COSMOmic method, included within COSMOperm, allows the calculation of partition coefficients of membranes or micelles, chemical potentials, and free energies of solutes in a layered system, which in turn makes it possible to obtain their distribution within the membrane [56,57,58,59,60]. The distribution of the different compounds that make up a biomembrane can be obtained using classic molecular dynamics simulations or using another extension of the COSMO-RS model, called COSMOplex. This extension generates divided liquid layers of variable composition as a representation of a biomembrane from information about its structure [61]. This makes it possible to generate a skin model with which COSMOperm works to calculate the resistance of the membranes, the permeability of individual compounds as well as their position in them, and the permeation pathway [46].

The computational skin model used in this work is based on the model proposed by Schwöbel et al. [46]. This model is based on the division of the outermost layer of the skin, the epidermis, into different compartments, or layers, with their own structure and cellular compositions [47], as can be seen in Table 2.

The skin penetration model is based on a series of resistances, *R*, from which the permeability coefficient, *K*_p_, can be calculated [62], as indicated in Equation (9).

(9) $$K_{p}=\frac{1}{R_{\mathrm{skin}}}$$

where *R*_skin_ is the overall skin resistance. To calculate *R*_skin_, it is first necessary to calculate the sum of all the resistances of each of the compartments that make up it, thus obtaining *R*_stratified-cells_ as indicated by Equation (10):

(10) $$R_{\mathrm{stratified}\text{-}\mathrm{cells}}=R_{\mathrm{SC}}+R_{\mathrm{SG}}+R_{\mathrm{SS}}+R_{\mathrm{SB}}$$

where *R*_SC_, *R*_SG_, *R*_SS_, and *R*_SB_ are the resistances of the SC, SG, SS, and SB compartments, described in Table 2, respectively. Starting from *R*_stratified-cells_ and also taking into account the shunt pathway, the *R*_skin_ is calculated following Equation (11):

(11) $$\frac{1}{R_{\mathrm{skin}}}=\frac{1}{R_{\mathrm{stratified}\text{-}\mathrm{cell}}}+\frac{1}{R_{\mathrm{shunt}}}$$

where *R*_shunt_ is the resistance of the shunt pathway and is kept constant 1/*R*_shunt_ = 2 × 10^−11^ m/s.

In addition, the transport of the compounds through the compartments is calculated by Equation (12):

(12) $$\frac{1}{R_{i}}=\frac{1}{R_{i,\mathrm{trans}}}+\frac{1}{R_{i,\mathrm{inter}}}$$

where i refers to the compartment (SC, SG, SS or SB), *R*_i,trans_ is the mechanism of transcellular absorption (through keratin-corneocytes by partitioning into and out of the cell membrane), and *R*_i,inter_ is the mechanism of intercellular absorption (trough corneocytes in the lipid-rich extracellular regions).

**Figure 3:**
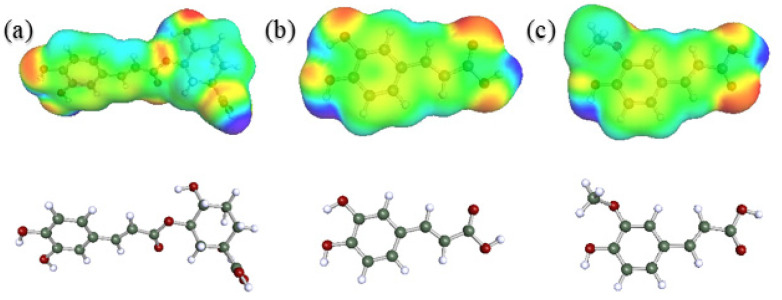
3D molecular structures of (**a**) CHA, (**b**) CAF, and (**c**) FA and their charge density (electronegative zone in red, electropositive zone in blue and neutral zone in green).

The skin model was applied by COSMO-RS, using the COSMOtherm software package for each of the tracked compounds in this work: CHA, CAF and FA. First, the pre-optimized 3D chemical structures from PubChem database were obtained, and then those structures were refined by using Gaussian version 9.0 with a DFT parametrization bvp86-TZVP (cartesian coordinates for optimized geometries can be found in the Supplementary Material). The 3D structures of the compounds and their surface charge density can be observed in Figure 3. The skin model, included in COSMOplex module, was used together with the COSMOperm extension to calculate the permeabilities of each compound as well as their position in the skin model and the resistances of each compartment.

## 3. Results and Discussion

### 3.1. SCE and Feed Solution Preparation

The pre-treated material, previously ground and sieved, was defatted in a first stage to facilitate the obtaining of the antioxidant fraction. This stage was usually carried out by means of maceration in hexane [41,45], but in a more recent study, supercritical extraction with CO_2_ was used [51]. This is due to the advantages offered by this type of extraction; since it is a non-toxic solvent, its polarity facilitates the extraction of lipophilic compounds and generates solvent-free final products because it can be easily removed by lowering the pressure. The supercritical extraction yield, *Y*_SCE_, was 8.3%, which is similar to the yields obtained by D. Baumman et al. (5.5–8.3%) [63] and A. Lopez-Padilla et al. (4.6–7.4%) [64] under comparable SCE conditions.

The polar fraction of *Calendula officinalis* was obtained by maceration in ethanol (EtOH) of the plant material after the SCE. The extraction yield obtained with this ethanolic maceration, *Y*_EtOH_, was 7.2%. Other authors [65] have obtained similar extraction yield (8.0%), although the plant material was not previously defatted and the proportional mass plant (g):EtOH (mL) was 1:6 instead of 1:10, as used in this work.

### 3.2. SAF Yields Statistical Analysis

Experimental values for *Y*_PV_ (wt%), *Y*_DV_ (wt%) and *Y*_SAF_ (wt%) for every experiment are gathered in Table 3. *Y*_PV_ varied from 12.3% (run 10; 120 bar-60 g/min) to 47.0% (run 1; 80 bar-35 g/min), whereas *Y*_DV_ oscillated between 1.3% (run 1; 80 bar-35 g/min) and 42.8% (run 12; 148 bar-53 g/min). It can be seen that at low pressures, PV yields are higher than DV yields, but this changes as the pressure increases; then, the yields equalize until *Y*_DV_ are greater than *Y*_PV_. The greatest difference was found in run 1, where *Y*_PV_ was 36 times the DV yield. Overall yields, *Y*_SAF_, varied between 31.1% (run 3; 92 bar-53 g/min) and 73.7% (run 12; 148 bar-53 g/min). As seen previously in other works [51], a full recovery of the entire mass of solutes contained in the feed solution is not possible due to the effect of two phenomena: the dragging of the most volatile components through the vent valve [66] and the deposition of materials in dead spaces.

The coefficients of Equation (8) were obtained for *Y*_PV_, *Y*_DV_ and *Y*_SAF_ and can be found in Table 4, along with their level of significance *p* and the coefficient R^2^ and the deviation s of the fitted mathematical model. The statistical analysis reveals that *Y*_PV_ depends on all terms (*β*_1_, *β*_2_, *β*_11_ and *β*_12_) except the quadratic term of CO_2_ (*β*_22_) and all of them are statistically significant (*p* < 0.05) except the pressure term (*β*_1_). *Y*_DV_ and *Y*_SAF_ depend on all terms being statistically significant only for *Y*_SAF_. For *Y*_DV_, the statistically significant terms are the pressure (*β*_1_), the quadratic term of pressure (*β*_11_), and the cross term (*β*_12_).

**Figure 4:**
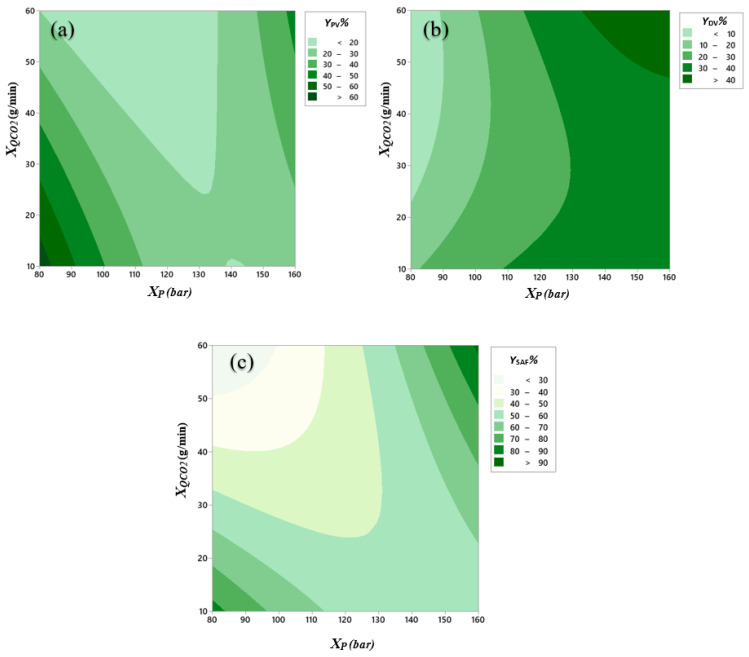
Contour plots of the yields: (**a**) at PV, *Y*_PV_%, (**b**) at DV, *Y*_DV_%, and (**c**) overall yield, *Y*_SAF_%, as function of pressure, *X*_P_ (bar) and CO_2_ flow rate, *X*_QCO_2__ (g/min).

In Figure 4, the contour plots corresponding to the surfaces defined by Equation (8) are shown for all yields (*Y*_PV_, *Y*_DV_ and *Y*_SAF_ as 1a, 1b and 1c, respectively). In Figure 4a, for flow rates above 12 g/min and below 24 g/min, *Y*_PV_ decreases as pressure increases, while for CO_2_ flow rates outside this range, *Y*_PV_ first decreases and then increases as pressure increases. For a fixed pressure, an increase in CO_2_ flow rate causes *Y*_PV_ to decrease except for high pressures, whereas the CO_2_ flow rate increases, *Y*_PV_ increases. For the studied ranges of pressure and CO_2_ flow rates, the highest *Y*_PV_ is found at low pressures (80–83 bar) and at low CO_2_ flow rates (10–16 g/min). Another maximum in *Y*_PV_ yield could be found at high pressures and CO_2_ flow rates (133–160 bar, 51–60 g/min). According to Figure 4b, *Y*_DV_ increases as pressure increases for a set CO_2_ flow rate. On the other hand, for a fixed pressure, a different behaviour is observed if the pressure is lower or higher than 133 bar: for low pressures, as the flow increases, *Y*_DV_ decreases, whereas the opposite effect is observed at higher pressures. In fact, the highest *Y*_DV_ is found at high pressures and CO_2_ flow rates (133–160 bar, 47–60 g/min). This may be due to a greater solubility of components and a higher dragging effect of the solutes towards DV. Analysing Figure 4c, it can be seen that *Y*_SAF_ follows a similar behaviour to *Y*_PV_, whereby the overall recovery of solutes from the feed solution is minimal at low pressures and high CO_2_ flow rates, whereas *Y*_SAF_ reaches a maximum when the two variables simultaneously reach their maximum (158–160 bar, 59–60 g/min) or minimum (80–83 bar, 10–12 g/min) values in the intervals considered.

### 3.3. Enrichment Ratio Analysis

In addition to the SAF global yields, ferulic acid (FA), caffeic acid (CAF) and chlorogenic acid (CHA) were monitored for the feed solution fraction (FS), precipitation vessel (PV) and downstream vessel (DV). The enrichment ratios *E*_i/j_ and *E*_ALL/PV_, defined by Equations (6) and (7), are gathered in Table 3 for all experiments performed. No *E*_i/DV_ is included, since the amount of none of the three compounds tracked (FA, CAF, CHA) was higher than the chromatographic detection limit, which indicates that they are mostly found in PV.

Only *E*_CHA/PV_, *E*_FA/PV_, and *E*_ALL/PV_ were correctly adjusted to Equation (8). The fitting coefficients of this equation are given in Table 4, where it can be seen that *E*_CHA/PV_ depends on all of the terms except the cross term (β_12_), and all of them are significant (*p* < 0.05) except for the linear term of the CO_2_ flow rate (β_2_). *E*_FA/PV_ and *E*_ALL/PV_ also depend on all terms except for the cross term (β_12_), but only β_0_ and the linear and quadratic pressure terms (β_1_, β_11_) are significant.

**Figure 5:**
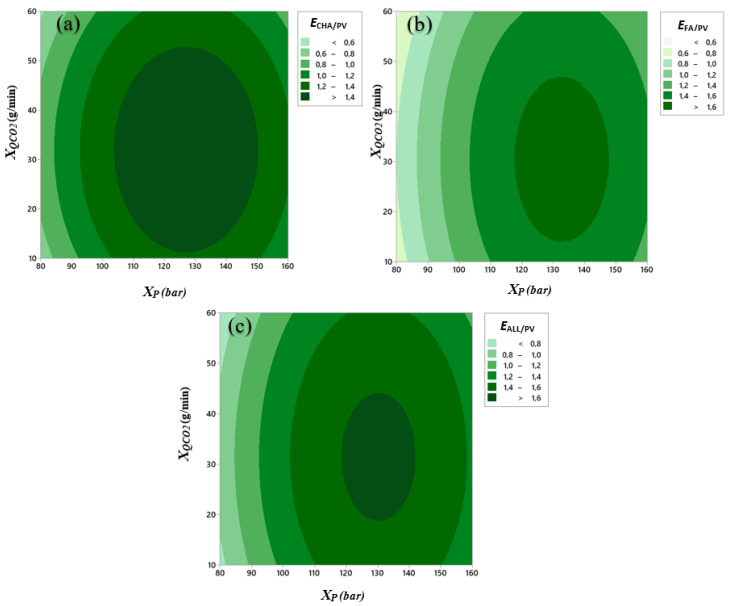
Contour plots of the enrichment ratios: (**a**) *E_CHA/PV_*; (**b**) *E_FA/PV_*, and (**c**) *E_ALL/PV_* as a function of pressure, *X*_P_ (bar), and CO_2_ flow rate, *X*_QCO_2__ (g/min).

Figure 5a–c shows the contour plot corresponding to the surface defined by Equation (8) for the *E*_CHA/PV_, *E*_FA/PV_, and *E*_ALL/PV_ enrichment ratios. As Figure 5a initially shows, *E*_CHA/PV_ increases as pressure increases and then decreases at higher pressure values. For a fixed pressure, *E*_CHA/PV_ behaves similarly with increasing CO_2_ flow rate. Then, the maximum is located in a central area, with pressure values between 103 and 150 bar and CO_2_ flow rates of 12–53 g/min. *E*_FA/PV_ and *E*_ALL_ have a similar behaviour, as can be seen from Figure 5b,c. The maximum of *E*_FA/PV_ is found for medium–high pressure values (117–147 bar) and intermediate CO_2_ flow rate values (14–47 g/min) within the considered intervals. For the enrichment of the three antioxidants, *E*_ALL/PV_, the maximum zone is found at intermediate values of both pressure and CO_2_ flow rate (118–142 bar, 18–44 g/min).

The fact that all of the analysed compounds, CHA, FA and CAF, are retained in PV makes the SAF technique perfect for obtaining a fraction enriched in these antioxidant compounds. Considering the precautions for experimental inaccuracies, the applied RSM model provides the upper limit of working conditions for achieving a significant enrichment of the compounds of interest. Optimum yield and enrichment values, provided by the applied RSM model, can be obtained working under conditions of 153 bar and 42 g/min.

In relation to the physical-mathematical treatment of the considered SAF process, it can be indicated that there are close similarities with the well-known supercritical antisolvent precipitation (SAS) processes suitable for the production of fine powders, or even composites including a bioactive substance and a polymer. However, the SAF modelling turns out to be much more complex, because mixtures consisting of a high number of components, such as the natural extracts studied in this work, are considered. Tentatively, most of the developments for the different steps implied in SAS could be adapted to SAF [32]. There are specialized papers about SAS that model both the whole process [67,68,69] as well as specific steps, such as breakup of the feed solution jet in the supercritical CO_2_ stream [70,71,72], mass transfer between droplets of organic solution and the compressed antisolvent [73,74,75], supersaturation and nucleation rate [76,77,78], growth mechanism [79], or the influence of the variables on precipitation [80]. However, as explained in a previous work [50], detailed modelling of the SAF process is quite a complex task, because of the high number of components in the extracts that usually need to be processed, then becoming rather inextricable from a rigorous theoretical study. In addition, reaction or association processes between the different components can occur. Therefore, and to the best of our knowledge, to date, there are no publications on the detailed theoretical modelling of SAF.

In any case, it seems clear that the different solubilities shown by the components of the mixture to be separated in the supercritical ethanol–CO_2_ mixture play an essential role in the fractionation process. In fact, it can be mentioned here that a very simple semi-empirical model containing the operating parameters (temperature and pressure) and the Hansen solubility parameters has been proved to be appropriate for describing the selectivity in a GAS-fractionation process closely related to SAF and SAS [81,82].

Related to the system considered in this work, it can be pointed out that the feed solutions present a glutinous character, which may be due to components such as resins or mucilage [83,84] not being completely solubilized. The presence of this solid material could induce a very effective process of heterogeneous nucleation [76]. This is how a practically complete precipitation in PV of the widely supersaturated CAF, CHA and FA occurs.

The optimal conditions for optimum yield and enrichment values, indicated above (153 bar, 42 g/min), are located between the extreme values of the ranges studied. This could be explained by the opposing effects that take place both for the increase in CO_2_ flow rate and pressure. An increase of CO_2_ flow rate implies a higher dragging effect, but also favours better mixing and nucleation. For its part, an increase in pressure would increase solubility, but favours nucleation of solutes.

### 3.4. Results of the Skin Model

The skin model applied was a fully hydrated skin model, and all the calculations for resistances, possible pathways and overall permeability values were performed using the COSMOtherm software package. The predicted values for Equations (9)–(11) for individual compounds are gathered in Table 5.

The compartment with the largest resistance for CAF and FA is the *stratum corneum* (log*R*_SC_ = −5.61 and −5.51 respectively), while for CHA, the largest resistance is the *stratum spinosum* (log*R*_SS_ = −7.06). This means that while for CAF and FA the *stratum corneum* is the main barrier in the penetration process, for CHA it manages to penetrate up to the *stratum spinosum*. Regarding the penetration pathway, both CAF and FA prefer the trans-corneocyte route (*R*_SC, inter_ >> *R*_SC, trans_), while CHA prefers the transcellular route of the *Stratum spinosum*, called the interstitial matrix (*R*_SC, inter_ << *R*_SC, trans_). The overall resistances, *R*_skin_, including the shunt pathway, are *R*_skin_ = *R*_skin_ = 7.74 × 10^7^, 1.86 × 10^9^ and 3.74 × 10^7^ for CAF, CHA and FA, respectively. Translating them using Equation (9), the permeability values for each compound were log*K*_p_ = −5.89 for CAF, log*K*_p_ = −7.27 for CHA and log*K*_p_ = −5.57 for FA. These values are corrected by a constant offset (Δlog*K*_p_ = −1.12 cm/s) [46], and the final permeability values are log*K*_p_ = −7.01, −8.39 and −6.69 for CAF, CHA and FA, respectively. Table 5 also shows the average calculated permeability values of CAF and FA in skin by Zhang et al. [85], and experimental permeability values from Caco−2 cells for CAF, CHA and FA [86,87], as well as the deviation of our corrected values log*K*_p_. It can be seen that our log*K*_p_ corrected values fit better to log*K*_p_ (calc) of skin by Zhang et al. (deviation = 0.16 and −0.46 for CAF and FA) than to the values of log*K*_p_ (exp) of Caco−2 cells (deviation = 1.17, 2.79 and 1.71 for CAF, CHA and FA respectively). This may be due to the fact that Caco−2 cell lines are used as a model of intestinal absorption and the estimation of oral bioavailability [88], and therefore, their permeability differs from the permeability of the skin. For a compound to be considered toxic, it must reach a certain layer of the skin. *Stratum corneum*, consisting mainly of dead cells, is considered a safe layer in which toxicity or irritation does not occur [89]. This makes both CAF and FA safe for cosmetic use. CHA, on the other hand, reaches a deeper layer of the epidermis, but does not reach the dermis, and furthermore, its permeability coefficient is lower than the desquamation rate coefficient (log*K*_D_ = −9) [90], which also makes it safe for cosmetic use. This desquamation rate coefficient indicates that even if a compound penetrates beyond the *stratum corneum*, the skin regenerates and prevents the compound from penetrating deeper in the epidermis [89].

## 4. Conclusions

The combination of two advanced separation techniques applied to *Calendula officinalis* flowers was successfully used to obtain extracts of interest in cosmetics. The initial supercritical extraction with CO_2_ (SCE) carried out, the yield of which was 8.3% and the subsequent maceration in ethanol applied, the yield of which was 8%, showed results similar to those obtained in works by other authors. Subsequently, the ability of the supercritical antisolvent fractionation (SAF) technique to fractionate the ethanolic extract to obtain an enriched fraction in the tracked compounds (CHA, FA, CAF) was satisfactorily evaluated through a Response Surface Methodology (RSM) based on Central Composite Design (CCD). Chlorogenic acid, ferulic acid and caffeic acid were obtained almost exclusively in the precipitated fraction, thus obtaining a fraction enriched in antioxidant compounds of great interest in the cosmetic industry and even with possible applications in the pharmaceutical or food industry. The CCD design also made it possible to deduce the pressure and CO_2_ flow rate conditions to obtain optimal yields and enrichment values were 153 bar and 42 g/min (composite desirability = 0.673).

Regarding the skin permeability model used, it was possible to evaluate the computational permeability of CAF, CHA and FA. In all cases, none of the compounds pass through the epidermis completely, which means that they are safe compounds for topical use on the skin. The final permeability values predicted by the model were log*K*_p_ = −7.01, −8.39 and −6.69 for CAF, CHA and FA, respectively and were compared with experimental values for both abdominal skin cells and Caco−2 intestinal cells, concluding that the skin model is a good model for predicting permeabilities and behaviours of compounds in the epidermis.

Although the SCE was carried out to favour obtaining the antioxidant phase, the extracts obtained through this technique are of great value due to their composition, and they are widely used in the cosmetic industry. In fact, a cosmetic formulation has been prepared containing extracts of *C. officinalis*, obtained under the conditions used in this work, that has been approved by the French agency ASNM (L’Agence nationale de sécurité du médicament et des produits de santé), and it is now being tested on human volunteers at MEDES (Institut de Médicine et de Physiologie Spatiales, Toulouse, France). All this makes these two advanced separation techniques (SCE and SAF), together with the experimental design and the skin permeability model used, powerful tools for obtaining, concentrating and evaluating compounds of interest to the cosmetic industry.

## Figures and Tables

**Table 1:** Codification and levels of the two independent variables considered in the factorial design of SAF experiments.

Variable	Symbol	Factor Levels				
		{−1.44	−1	0	1	1.44}
Pressure (bar)	*X*P	80	92	120	148	160
CO_2_ flow rate (g/min)	*XQ*_CO__2_	10	17	35	53	60

**Table 2:** Compartments of epidermis, their abbreviations, and descriptions of each layer.

Compartment	Abbreviation	Description
Stratum corneum	SC	Horny layer, the outermost layer and the main barrier to permeability within the skin
Stratum granulosum	SG	Granular layer
Stratum spinosum	SS	Spinous or prickle layer, release neutral barrier lipids
Stratum basale	SB	Basal layer, metabolically active
Appendageal compartment	Shunt	Responsible for transport of chemical through the hair follicles sweat glands, and sebaceous glands

**Table 3:** Operational conditions of pressure, *X*_P_, and CO_2_ flow rate *XQ*_CO_2__, for every run of the CCD design of the SAF process as well as the corresponding results for the yields and enrichment ratios, defined by Equations (3)–(7).

Run	Exp. Run Order	*X*_P_ (bar)	*XQ*_CO_2__(g/min)	*Y*_PV_ (wt%)	*Y*_DV_ (wt%)	*Y*_SAF_ (wt%)	*E*_CHA/PV_	*E*_CAF/PV_	*E*_FA/PV_	*E*_ALL/PV_
1	9	80	35	47.0	1.3	48.3	0.86	1.19	0.69	0.79
2	4	92	17	41.0	21.5	62.5	1.04	1.25	1.10	1.09
3	10		53	16.1	15.0	31.1	1.08	1.33	1.16	1.21
4	13	120	10	26.0	32.2	58.2	1.51	1.48	1.67	1.64
5	2		35	26.1	20.3	46.4	1.53	1.36	1.55	1.52
6	3		35	22.0	25.8	47.8	1.52	1.37	1.54	1.52
7	6		35	18.5	30.0	48.5	1.40	1.44	1.56	1.66
8	7		35	15.1	30.5	45.6	1.59	1.40	1.73	1.66
9	11		35	13.6	29.6	43.2	1.72	1.39	1.70	1.67
10	12		60	12.3	30.4	42.7	1.14	1.30	1.25	1.27
11	8	148	17	23.4	31.3	54.7	1.18	1.40	1.32	1.32
12	5		53	30.9	42.8	73.7	1.33	1.41	1.52	1.46
13	1	160	35	31.1	34.3	65.4	1.28	1.37	1.55	1.46

**Table 4:** Fitting coefficients of Equation (8) for PV, *Y*_PV_, DV, *Y*_DV_, and overall, *Y*_SAF_, yields, chlorogenic and ferulic acid enrichment ratio in PV (*E*_CHA/PV_, *E*_FA/PV_ respectively) total enrichment ratio (*E*_ALL/PV_) as well as the significance of each term, *p*, the regression coefficients, R^2^, and standard deviation, s.

	*Y*_PV_/wt%		*Y*_DV_/wt%		*Y*_SAF_/wt%		*E*_CHA/PV_		*E*_FA/PV_		*E*_ALL/PV_	
	Coefficient Value	*p*	Coefficient Value	*p*	Coefficient Value	*p*	Coefficient Value	*p*	Coefficient Value	*p*	Coefficient Value	*p*
*β*_0_	284.3	0.000	−40.3	0.000	242.6	0.000	−3.889	0.000	−4.11	0.000	−3.840	0.000
*β*_1_	−3.594	0.075	1.241	0.000	−2.343	0.000	0.0796	0.026	0.0835	0.003	0.0802	0.004
*β*_2_	−2.204	0.019	−1.691	0.833	−3.835	0.003	0.0253	0.381	0.0160	0.454	0.0166	0.479
*β*_11_	0.01215	0.000	−0.00493	0.035	0.00717	0.001	−0.000314	0.001	−0.000315	0.002	−0.000308	0.001
*β*_22_	-	-	0.00898	0.105	0.00812	0.040	−0.000395	0.034	−0.000262	0.189	−0.000264	0.125
*β_1_*_2_	0.01620	0.006	0.00900	0.058	0.02520	0.000	-	-	-	-	-	-
R^2^	88.28	-	91.26	-	96.74	-	83.00	-	82.91	-	84.54	-
s	4.42	-	4.00	-	2.66	-	0.13	-	0.15	-	0.13	-

**Table 5:** Detailed model parameters of resistances and and *K*_p_ (predicted, calculated, experimental and their deviations) for CAF, CHA and FA for each compartment and their permeation path.

Parameter	Caffeic Acid	Chlorogenic Acid	Ferulic Acid
Vehicle	water	water	water
Skin membrane	epidermis	epidermis	epidermis
Rate limiting step	SC via polar trans-corneocyte pathway	SS via interstitial space	SC via polar trans-corneocyte pathway
log*K*_vehicle:water_	0.00	0.00	0.00
*R*_SC,inter_ (s/m)	2.15 × 10^12^	8.20 × 10^16^	2.55 × 10^11^
*R*_SC,trans_ (s/m)	4.07 × 10^7^	1.76 × 10^8^	3.23E × 10^7^
*R*_SC_ (s/m)	4.07 × 10^7^	1.76 × 10^8^	3.23E × 10^7^
Log*R*_SC_ (cm/s)	−5.09	−6.71	−4.21
*R*_SG,inter_ (s/m)	1.26 × 10^8^	5.14 × 10^8^	1.23 × 10^8^
*R*_SG,trans_ (s/m)	1.34 × 10^7^	5.13 × 10^11^	1.63 × 10^6^
*R*_SG_ (s/m)	1.22 × 10^7^	5.13E × 10^8^	1.61 × 10^6^
Log*R*_SG_ (cm/s)	−5.32	−7.06	−4.46
*R*_SS,inter_ (s/m)	2.86 × 10^8^	1.17 × 10^9^	2.80 × 10^8^
*R*_SS,trans_ (s/m)	2.26 × 10^7^	8.54 × 10^11^	2.91 × 10^6^
*R*_SS_ (s/m)	2.09 × 10^7^	1.16 × 10^9^	2.88 × 10^6^
Log*R*_SS_ (cm/s)	−4.57	−5.91	−3.80
*R*_SB,inter_ (s/m)	1.98 × 10^7^	8.07 × 10^7^	1.94 × 10^7^
*R*_SB,trans_ (s/m)	4.58E × 10^6^	1.71 × 10^11^	6.50 × 10^5^
*R*_SB_ (s/m)	3.72 × 10^6^	8.06 × 10^7^	6.29 × 10^5^
Log*R*_SB_ (cm/s)	−5.89	−7.27	−5.57
*R*_cells_ (s/m)	7.75 × 10^7^	1.93 × 10^9^	3.74E × 10^7^
*R*_shunt_ (s/m)	5.00 × 10^10^	5.00 × 10^10^	5.00 × 10^10^
*R*_skin_ (s/m)	7.74 × 10^7^	1.86 × 10^9^	3.74E × 10^7^
log*K*_p_ (pred.) (cm/s)	−5.89	−7.27	−5.57
log*K*_p_ (pred.) + cte offset (cm/s)	−7.01	−8.39	−6.69
log*K*_p_ (calc.) epidermis (cm/s)	−6.85	-	−7.15
Deviation	0.16	-	0.46
log*K*_p_ (exp.) Caco−2 (cm/s)	−5.84	−5.60	−4.98
Deviation	1.17	2.79	1.71

## Data Availability

Data is contained within the article and the Supplementary Materials.

## Supplementary Materials

The following are available online at https://www.mdpi.com/article/10.3390/antiox11010096/s1, Info S1: Cartesian Coordinates for Optimized Geometries in Gas Phase at bvp86.
