# Supplementary material for: Concentration of Antioxidant Compounds from Calendula officinalis through Sustainable Supercritical Technologies, and Computational Study of Their Permeability in Skin for Cosmetic Use
Source: Antioxidants (Basel). 2021 Dec 30;11(1):96. doi: 10.3390/antiox11010096 (PMC8773024; doi:10.3390/antiox11010096)
Supplement: Supplementary file 1 [file antioxidants-11-00096-s001.zip › antioxidants-1500105-supplementary.pdf]

Concentration of antioxidant compounds from Calendula officinalis through sustainable supercritical technologies and computational study of their permeability in skin for cosmetic use

Raquel Mur [1], Elisa Langa [2], M. Rosa Pino-Otín [2], José S. Urieta [1] and Ana M. Mainar [1,*]

[1]GATHERS Group, Aragón Institute of Engineering Research (I3A), Universidad de Zaragoza, c/. Mariano Esquillor s/n, 50018 Zaragoza, Spain; 649396@unizar.es (R.M.), urieta@unizar.es (J.S.U.)

[2]Universidad San Jorge, Campus Universitario Villanueva de Gállego, Autovía A-23 Zaragoza-Huesca km. 299, 50830 Villanueva de Gállego (Zaragoza), Spain; elanga@usj.es (E.L.), rpino@usj.es (M.R.P.O.)

[*]Correspondence: ammainar@unizar.es; Tel.: +34 976761195

.

Supplementary Material

Cartesian Coordinates for Optimized Geometries in Gas Phase at bvp86

Caffeic acid

O	-3.32883600	1.80238700	0.00013600
O	-4.19457800	-0.69392800	-0.00017100
O	4.70919500	-0.86691600	0.00007800
O	4.04035800	1.31003200	-0.00013200
C	-0.07419000	0.03446400	0.00003700
C	-1.04516600	1.05782300	0.00003800
C	-0.55206800	-1.29128200	0.00002600
C	-2.40918200	0.80269900	0.00003600
C	-2.83082500	-0.54479300	0.00000400
C	-1.91092100	-1.59259100	0.00001500
C	1.33566400	0.36029800	-0.00006800
C	2.41177000	-0.44933300	0.00003000
C	3.76633400	0.12356600	-0.00000500
H	-0.71966600	2.09709000	0.00008500
H	0.15995500	-2.11337000	0.00006100
H	-2.25308600	-2.62382600	0.00002300
H	1.56524900	1.42994000	-0.00017600
H	2.32117400	-1.53368700	0.00010900
H	-4.19465900	1.34245500	-0.00017900
H	-4.39071400	-1.64909400	0.00007700
H	5.55413000	-0.36721400	0.00004300

Ferulic acid

O	-2.59988000	1.65394300	-0.42433300
O	-3.92502100	-0.77927000	-0.19180700
O	4.67859400	1.41444200	-0.09957800
O	4.56793600	-0.84445400	0.16655500
C	0.27087200	-0.64324100	0.00383700
C	-1.91634600	0.48572200	-0.18700100
C	-0.53212700	0.51038200	-0.13815600
C	-0.42203200	-1.86119400	0.11313400
C	-2.56392400	-0.77353200	-0.09726500
C	-1.80975700	-1.93891900	0.06886300
C	1.71514800	-0.60651900	0.04653200
C	2.55634400	0.44079700	-0.04981100
C	-3.60534000	1.99494400	0.55539000
C	4.00998600	0.22821800	0.02118000
H	-0.06001000	1.48827000	-0.21779500
H	0.14826200	-2.78044200	0.23256400
H	-2.31259200	-2.90002100	0.14295700
H	2.19904100	-1.57887200	0.17648900
H	2.19840200	1.45970300	-0.18329600
H	-3.13827300	2.24081200	1.51500300
H	-4.32552200	1.18292700	0.69079500
H	-4.11335000	2.87722900	0.16345600
H	-4.20638900	-1.71118800	-0.10804700
H	5.62046600	1.14433900	-0.03903100

Cartesian Coordinates for Optimized Geometries in Gas Phase at bvp86

Chlorogenic acid

O	0.75915900	-0.88140600	-0.27476600
O	4.29987400	0.81466400	1.35898200
O	2.32011700	-3.06830500	0.16949600
O	4.56225200	-1.84324700	1.44728400
O	4.72083900	2.09675300	-1.95490400
O	5.46278000	2.74142000	0.09395400
O	0.13667900	0.76762400	1.18983400
O	-7.30653000	1.53018300	0.99813900
O	-8.07913800	-0.27640100	-0.75779400
C	4.19167100	0.71494600	-0.06291300
C	2.14244900	-0.65658700	0.13801600
C	2.70466900	0.63408700	-0.44503100
C	4.95963900	-0.52459300	-0.58453300
C	2.88717400	-1.88796800	-0.35456400
C	4.36737900	-1.79625300	0.05040100
C	4.85293400	1.96559400	-0.62235100
C	-0.16252500	-0.08117000	0.37171000
C	-1.51074600	-0.41664900	-0.09985600
C	-2.58364100	0.24063300	0.38561000
C	-3.98516300	0.08481000	0.07485100
C	-4.95200600	0.89961900	0.70695800
C	-4.47716500	-0.85740400	-0.85134000
C	-6.30314000	0.78354900	0.43505000
C	-5.82718600	-1.00101700	-1.14788800
C	-6.75104300	-0.17436400	-0.50108800
H	2.16765100	-0.61568000	1.23356800
H	2.60416500	0.62967700	-1.53464000
H	2.16411500	1.49312600	-0.03827600
H	6.01775300	-0.43765700	-0.31764800
H	4.88864800	-0.56770600	-1.67473200
H	2.84830100	-1.88305700	-1.46173300
H	4.87241600	-2.67581300	-0.37439400
H	4.92909000	1.57131300	1.47081500
H	1.35775700	-2.90989700	0.08787500
H	4.53131100	-0.89789300	1.72980300
H	5.21099700	2.92033900	-2.17162900
H	-1.60316400	-1.20386900	-0.84454700
H	-2.35534600	1.01052900	1.12858900
H	-4.61911200	1.64374100	1.43126700
H	-3.77033300	-1.50789900	-1.36262000
H	-6.17910900	-1.73515500	-1.86467100
H	-6.89522500	2.15066500	1.62786300
H	-8.49797300	0.40156000	-0.18488000
